# Effects of oleoylethanolamide supplementation on atherogenic indices and hematological parameters in patients with nonalcoholic fatty liver disease: A clinical trial

**DOI:** 10.34172/hpp.2020.56

**Published:** 2020-11-07

**Authors:** Helda Tutunchi, Fatemeh Naeini, Maryam Saghafi-Asl, Nazila Farrin, Alireza Monshikarimi, Alireza Ostadrahimi

**Affiliations:** ^1^Nutrition Research Center, Department of Clinical Nutrition, School of Nutrition and Food Sciences, Tabriz University of Medical Sciences, Tabriz, Iran; ^2^Department of Clinical Nutrition, School of Nutritional Sciences and Dietetics, Tehran University of Medical Sciences, Tehran, Iran

**Keywords:** Atherogenic indices, Hematological parameters, Non-alcoholic fatty liver disease, Oleoylethanolamide, PPAR alpha

## Abstract

**Background:** Non-alcoholic fatty liver disease (NAFLD) is the most frequent cause of chronic liver disease in the world. The current interventional trial aimed to evaluate the effects of supplementation with oleoylethanolamide (OEA) in combination with weight loss intervention on some atherogenic indices as well as hematological parameters in patients newly diagnosed with NAFLD.

**Methods:** In this triple-blinded, randomized, placebo-controlled clinical trial, 76 obese patients with NAFLD confirmed by ultra-sonographic findings were randomly assigned to receive a weight reduction diet plus either 250 mg OEA (n=38) or placebo (n=38) for 12 weeks. Atherogenic factors including total cholesterol/high-density lipoprotein cholesterol (HDL-C),low-density lipoprotein cholesterol (LDL-C)/HDL-C, triglyceride (TG)/HDL-C, non-HDL-C/HDL-C ratios and non-HDL-C level, as well as hematological parameters were assessed before and after intervention.

**Results** : After adjustment for potential confounding factors, between group analyses demonstrated a significantly lower LDL-C/HDL-C, TG/HDL-C, and non-HDL-C/HDL-C ratios in the OEA group compared to the placebo, post-intervention (95% confidence interval [CI]:0.06 to 0.85, P = 0.024; 95% CI: -2.06 to -0.05, P = 0.039; 95% CI: -1.05 to -0.02, P = 0.042,respectively). Additionally, OEA supplementation could significantly decrease the levels of red blood cell distribution width (RDW) compared to the placebo at the endpoint after considering potential confounding variables (95% CI: -0.56 to -0.003, P = 0.041). No significant differences were found between the two study groups in terms of other hematological parameters.

**Conclusion:** The results of the current study indicated that OEA supplementation had beneficial effects on LDL-C/HDL-C, TG/HDL-C, and non-HDL-C/HDL-C ratios as well as RDW in obese patients with NAFLD.

**Trial Registration:** IRCT20110530006652N2; https://www.irct.ir/trial/37228.

## Introduction

Non-alcoholic fatty liver disease (NAFLD), caused by accumulation of triglyceride (TG) in the cytoplasm of hepatocytes, is regarded as one of the most common liver pathologies worldwide.^1^ NAFLD encompasses a wide range of liver disorders including simple hepatic steatosis, non-alcoholic steatohepatitis (NASH), fibrosis, cirrhosis and eventually hepatocellular carcinoma, with a complex ‘multi-hit’ pathophysiology.^2^ Although the real prevalence of NAFLD may vary depending on the sensitivity of the detection method used, there has been a marked elevation in the prevalence of NAFLD in recent years.^3,4^ It has been projected that this disease will affect 33.5% of the adult population by 2030, of which 27% will suffer from NASH. The disease is remarkably correlated with obesity, insulin resistance (IR), type 2 diabetes, hypertension, and hyperlipidemia.^2,5,6^ NAFLD is characterized by an atherogenic lipid profile including increased TG, low-density lipoprotein cholesterol (LDL-C), and very low-density lipoprotein cholesterol (VLDL-C) concentrations and a reduced high-density lipoprotein cholesterol (HDL-C) level leading to a significantly increased risk of cardiovascular disease (CVD).^7-9^ It has also been suggested that total cholesterol (TC)/HDL-C, LDL-C/HDL-C, and TG/HDL-C ratios are better mirror of the metabolic and clinical interactions between lipid fractions and have higher predictive values than isolated parameters used independently in disease states.^10^ Furthermore, non-HDL-C which reflects all of the major lipoproteins associated with a higher risk of CVD may better identify abnormal lipid profile in patients with NAFLD.^11,12^ Recent evidence also suggests that the non-HDL-C/HDL-C ratio is an independent predictor of NAFLD.^13,14^ Oxidative stress (OS) also has an important role in the pathogenesis and severity of NAFLD.^15^ Moreover, red blood cell (RBC) destruction and death are finally caused by OS. Impaired RBC deformability was found in pathological conditions associated with increased OS. In disease states, reactive oxygen species (ROS)-mediated damage of erythrocyte membrane components appears to increase the rigidity and fragility of RBC membrane.^16^ In addition, NAFLD was associated with red blood cell distribution width (RDW) and NAFLD patients were more likely to have high levels of RDW, which has been reported to be significantly related to CVD morbidity and mortality.^17,18^

Previous studies have demonstrated that lifestyle interventions, principally dietary modification and regular exercise, are the cornerstones of NAFLD treatment.^19,20^ In addition, it has been suggested that peroxisome proliferator-activated receptor (PPAR) agonists ameliorate metabolic disorders, inflammation, and OS related to NAFLD. Therefore, these drugs are attractive targets to tackle NAFLD.^21-23^ Fibrates, low-affinity ligands for PPAR‐α receptors, are recommended in patients with NAFLD. However, these drugs have dose‐dependent side effects.^24^ Therefore, novel PPARα agonists with greater potency and efficacy may be more useful in the management of NAFLD. ^21^ There has been growing interest in oleoylethanolamide (OEA), which binds to PPAR-α with high affinity^21^. The main dietary sources of OEA include oatmeal, nuts, and cacao powder. Moreover, dietary oleic acid acts as the main substrate for OEA production. ^22^ Furthermore, it has been shown that treatment with OEA as a pharmacologic drug can reduce appetite and induce lipolysis and fatty acid (FA) oxidation.^22,25,26^ Anti-inflammatory, antioxidant, atheroprotective, and anti-atherosclerosis effects of OEA have also been demonstrated in animal studies.^27-30^ It is well documented that the biological effects of OEA are primarily mediated via activation of PPAR-α signaling pathway.^31,32^ PPARα is a key regulator of lipid metabolism by elevating tissue-specific expression of its target genes involved in FA uptake and beta-oxidation.^33^ Considering the lack of randomized clinical trial assessing the impacts of OEA administration on atherogenic indices and hematologic parameters in patients with NAFLD, the present study aimed to investigate the impacts of OEA treatment in combination with calorie restriction on some atherogenic indices and hematological parameters in these patients.

## Materials and Methods

### Study design

This triple-blinded, randomized, placebo-controlled clinical trial was performed between March to September 2019 in the northwest of Iran. Following a public announcement of the study, subjects were enrolled from the polyclinics and healthcare centers in the northwest of Iran. NAFLD was diagnosed through ultrasonography using a SonoAce X4 ultrasound system (Medison Inc., Korea) after overnight fasting. Before enrollment, written, informed consent was taken from the patients. The study protocol was approved by the Ethics Committee of the Tabriz University of Medical Sciences, which was in compliance with the Helsinki Declaration. This trial was registered in the Iranian Registry of Clinical Trials (Identifier: IRCT20110530006652N2). The reporting of this work is compliant with STROBE guidelines.

### Participants

Seventy-six obese, newly diagnosed NAFLD patients (body mass index [BMI]: 30-40 kg/m^2^) aged 21-59 years were involved. Patients with liver diseases such as hepatitis, cirrhosis, biliary disorders, inherited disorders affecting liver, those with diabetes, hypertension, cardiovascular disorders, kidney dysfunctions, thyroid problems, gastrointestinal disorders, pulmonary and autoimmune disease, malignancies, and recent surgery, and those who were alcoholic, smoker and tobacco consumer were excluded. In addition, exclusion criteria were as follows: pregnancy or lactation, under weight-loss programs within three months prior to the study, use of lipid-lowering drugs, weight loss drugs, corticosteroids, hepatotoxic drugs, anticoagulants, antidiuretics, multivitamins, minerals, and any dietary supplements during the past three months.

### Sample size 

Considering confidence interval (CI) of 95%, a power of 90%, and a two-tailed statistical test, the sample size was calculated to be 34 for each group, based on the mean and standard deviation of PPAR-α, obtained from a former trial conducted by Payahoo et al^34^ anticipating an approximate drop-out rate of 10% during the study period, 38 subjects were recruited in each group.

### Randomization and intervention 

Eligible patients were randomized to receive either OEA or placebo for 12 weeks based on the random block procedure developed by random allocation software. Stratification was done for sex and severity of NAFLD (grade 1 vs. grade 2). The random sequence was administered by an independent third investigator who was not aware of the study clinical process until the outcome data collection was completed. Patients in the both OEA and placebo group was given two capsules per day for 12 weeks. OEA group received capsules which contained 125 mg OEA, while placebo group received capsules which contained 125 mg starch. All participants were asked to take the capsules 30 minutes before their main meals. All capsules were similar in appearance, size, and color. Both OEA and placebo capsules were labeled as A or B, and the investigators, participants, and the statistician were blinded to the drug allocation until the end of the analysis. The OEA capsules were provided by Nutrition Research Center of Tabriz University of Medical Sciences. All participants were monitored every two weeks to check the compliance and probable side effects. The patients were asked to get back the residual capsules at each visit for determination of compliance. Missing more than 10% of the capsules was considered incompliance.

All patients were given weight loss diet according to published recommendations. A 3-day food record was obtained from each participant to evaluate adherence to dietary advice. In addition, physical activity (PA) recommendations were given to all the patients. They were recommended to exercise for 30 minutes at least three days a week.

### Physical activity and blood pressure assessment

To assess PA levels of the participants, a validated international PA questionnaire short form (IPAQ-SF) was applied. A trained researcher completed the questionnaire by a face-to-face interview. Based on the scoring method of IPAQ, metabolic equivalents (MET-min/wk) scores were computed, and the PA level was reported as high (>3000 MET), moderate (600-3000 MET), or low (<600 MET) activity.^35^ Additionally, after at least 5 minutes of rest, blood pressure measurement was done with aneroid sphygmomanometer and stethoscope in a comfortable sitting position.

### Laboratory analysis

Laboratory assessment included complete blood count and lipid profile. Venous blood samples were obtained after an overnight fasting and a part of the venous blood samples centrifuged to extract serum. Serum TC, TG, HDL-C, and LDL-C concentrations were assessed by the enzymatic colorimetric method, by commercial kits (Pars Azmoon Co., Tehran, Iran). TC/HDL-C, LDL-C/HDL-C, and TG/HDL-C ratios were also computed. Non-HDL-C was computed as TC minus HDL-C and then non-HDL-C/HDL-C ratio was determined. Full blood count including red cell count, hemoglobin (Hb), white cell count, platelets as well as Hb indices were assessed from the remaining whole blood that was placed in EDTA tubes using Hematology Analyzer (Nihon Kohden Celltac alpha MEK-6510).

### Statistical analysis

All statistical analyses were done using IBM SPSS Statistics version 21 (IBM SPSS Statistics, IBM Corp, ARMONK, USA). We used Kolmogorov-Smirnov test to assess the normal distribution of the data. Quantitative and qualitative variables were presented as mean (standard deviation) and frequency (percentage), respectively. Independent samples *t* test and Fisher’s exact test were applied to assess the differences between the study groups at baseline. Paired samples *t* test was used for assessing within-group changes. Analysis of covariance (ANCOVA) was run to compare the two groups at the end of the research adjusted for baseline values and confounding variables. All analyses were controlled for BMI, age, changes in PA level, and energy intake. *P* value less than 0.05 was considered statistically significant.

## Results

**Figure 1:**
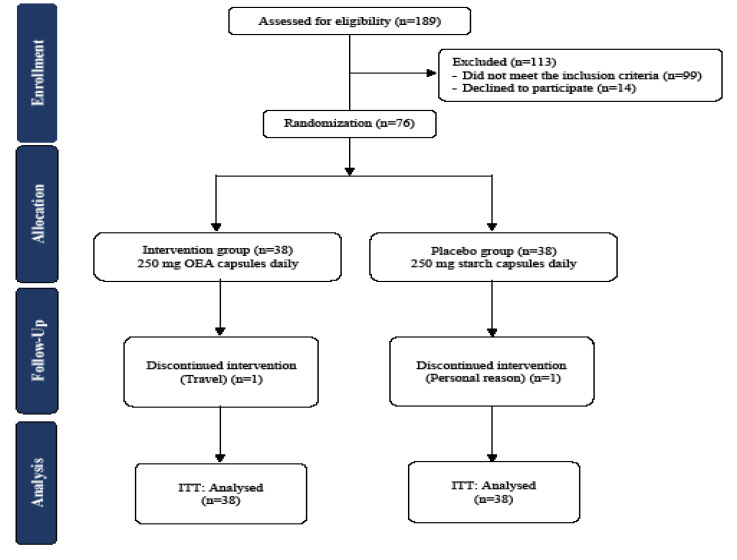
Flowchart of the study

In the current clinical trial, all variables were normally distributed. A total of seventy-six patients with NAFLD were recruited in the present research. However, seventy-four patients completed the trial. There was one drop-out in each group for personal reasons (Figure 1). The analysis was done based on the intention-to-treat approach. Therefore, all patients (n=76) were included in the final analysis. No major adverse events were found following supplementation in the study groups.

Table 1 presents the baseline characteristics of the participants. The mean age of the patients in the OEA and placebo group was 41.73 (6.95) and 39.27 (7.14), respectively. At baseline, no significant difference was found between the two groups in terms of serum TC, TG, LDL-C, HDL-C, fatty liver severity and the levels of PA.

As shown inTable 2, serum LDL-C/HDL-C and non-HDL-C/HDL-C concentrations decreased significantly in both study groups, while TC/HDL-C, TG/HDL-C, and non-HDL-C decreased significantly only in the OEA group (*P* <0.01). After adjustment for potential confounders, between group analyses demonstrated a significantly lower LDL-C/HDL-C, TG/HDL-C, and non-HDL-C/HDL-C ratios in the OEA group compared to the placebo, post-intervention (95% CI: 0.06 to 0.85, *P* = 0.024; 95% CI: -2.06 to -0.05, *P* = 0.039; 95% CI: -1.05 to -0.02, *P* = 0.042, respectively). As presented in Table 2, systolic blood pressure reduced significantly in both OEA and placebo groups; although, it was not significantly different between the groups at the end of the study. No significant within- and between-group differences were observed for diastolic blood pressure and PA level.

Table 3 shows hematological factors of the study participants. At the end of the study, Hb, hematocrit (HCT), mean corpuscular volume (MCV), mean corpuscular hemoglobin (MCH), and mean corpuscular hemoglobin concentration (MCHC) increased significantly in both study groups, while RBC increased significantly only in the OEA group (*P* = 0.004). Post-intervention, RDW, platelet (PLT) number, and platelet distribution width (PDW) decreased significantly in both study groups. In addition, OEA supplementation could significantly decrease the levels of RDW in comparison with the placebo at the endpoint after considering potential confounding variables (95% CI: -0.56 to -0.003, *P* = 0.041). However, no significant differences were observed between the study groups, in terms of other hematological parameters.

## Discussion

**Figure 2:**
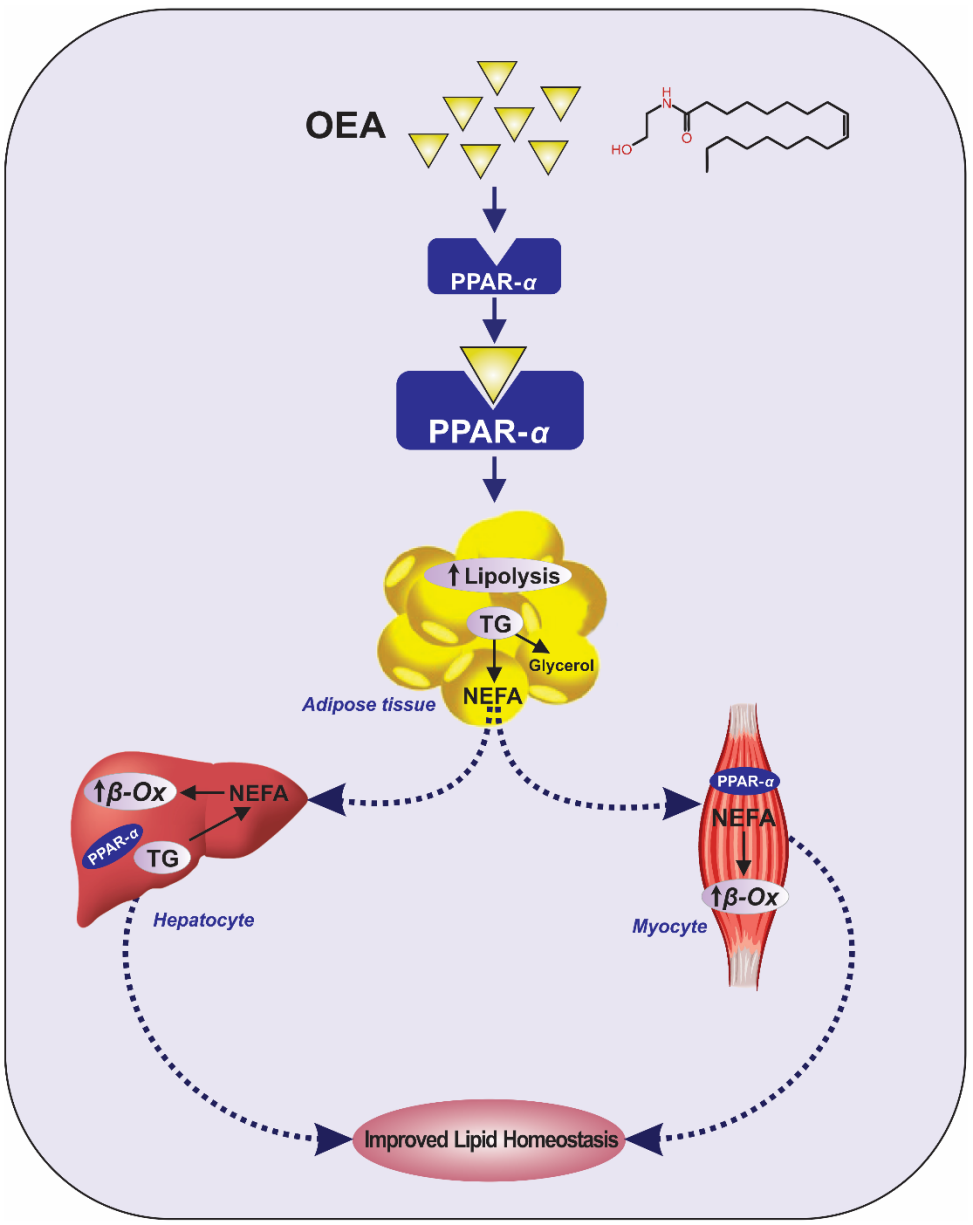
PPossible mechanisms of OEA actions in improving lipid profile. Activation of PPAR-α by OEA in the adipocyte increases triglyceride lipolysis, releasing fatty acids from the adipocyte for uptake in oxidative tissues, especially muscle cells and hepatocytes. β-Ox, Beta-oxidation; NEFA, Non-esterified fatty acids; OEA, Oleoylethanolamide; PPAR-α, Peroxisome proliferator-activated receptor-α; TG, Triglyceride

For the first time, the present randomized, triple-blind trial assessed the impacts of calorie restriction plus OEA administration on the atherogenic indices and hematological parameters in NAFLD patients. The main finding of the current trial was that treatment with OEA, an endogenous PPAR-α ligand, resulted in a significant decrease in some atherogenic parameters including LDL-C/HDL-C, TG/HDL-C, and non-HDL-C/HDL-C ratios in the OEA group, compared to the placebo group after considering potential confounding variables. Although TC/HDL-C ratio and non-HDL-C level decreased significantly in the OEA group, no statistically significant differences were found between the two groups, post-intervention. Previous studies have demonstrated that TC/HDL-C, LDL-C/HDL-C, and TG/HDL-C ratios are better indicators of CVD, which is one of the most major causes of morbidity and mortality in patients with NAFLD.^36-40^ The mechanism by which OEA exerts beneficial metabolic effects is mainly attributed to PPAR-α.^21,26^ Activation of PPAR-α by OEA and the consequential transcription of PPAR-α modulated genes lead to a cascade of events that finally affects metabolic parameters.^26^ PPAR-α is highly expressed in tissues with high rates of FA oxidation including liver, heart, skeletal muscle, and brown adipose.^41^ PPAR agonists have been widely used for decades as lipid-lowering agents.^23^ Possible mechanisms of action of OEA in improving the lipid profile is shown in Figure 2.

Mechanistically, OEA activates PPAR-α receptor and PPAR-α modulates the expression of genes encoding proteins that are involved in lipid metabolism such as uncoupling protein 2, fatty acid translocase/cluster of differentiation 36 (FAT/CD36), and fatty acid binding protein, thereby improving atherogenic dyslipidemia which is strongly associated with the prevalence of NAFLD.^21,22^ The lipid-lowering effects along with atheroprotective functions are the mechanisms underlining the anti-atherosclerosis action of the OEA.^27^ Overall, our results were concordant with the findings of murine studies in terms of atherogenic factors. In vivo rodent models indicated that OEA administration could enhance lipid utilization via stimulating FA uptake, intracellular transport, intracellular lipolysis, and fat oxidation, and could also regulate lipid levels in tissue and circulation.^25,26,32^ Li et al^42^ assessed the effects of OEA supplementation in high fat diet-induced NAFLD rats. They found that the intraperitoneal (IP) administration of 5 mg/kg OEA could significantly reduce TC and TG levels in the treatment group compared to the control group. In the same study, OEA treatment significantly elevated the mRNA expression levels of genes involved in FA beta-oxidation in the liver, including PPAR-a, carnitine palmitoyltransferase I, and mitochondrial short-chain enoyl-CoA hydratase 1 (ECHS1) in a PPAR-a-dependent way.^42^ Results from animal studies also indicated that the administration of OEA could significantly decrease both serum cholesterol and TG concentrations following one,^43^ two,^25,31^ and four weeks.^44^

Regarding hematological factors, there are studies demonstrating that function and morphology of platelets are altered in patients with diabetes and metabolic syndrome.^45,46^ Furthermore, a growing body of evidence suggests that hematological abnormalities may also occur in patients with NAFLD. For example, NAFLD patients significantly had higher circulating Hb concentration than healthy subjects.^47^ In some previous studies, higher values of RDW and PDW were also observed in NAFLD patients, compared to controls.^17,18,48^ Additionally, several independent studies have found that steatosis was associated with higher MPV.^49,50^ Based on our expansive search, there were no previous studies examining the effects of OEA on hematological factors. In our study, OEA supplementation could significantly decrease RDW. RDW is a parameter that reflects the heterogeneity of erythrocyte volume.^51^ Higher RDW was found to be a risk marker of morbidity and mortality for CVD in various study populations.^52^ In a study by Yang et al,^17^ the RDW was significantly increased in NAFLD patients. Furthermore, Kim et al^18^ reported that higher RDW was independently correlated with advanced fibrosis in NAFLD even after adjusting for confounding variables. A significant positive correlation was also reported between RDW and inflammatory markers in previous studies.^53^ On the other hand, a large number of studies suggest that NAFLD is strongly associated with inflammation and pro-inflammatory cytokines were significantly elevated in NAFLD, which may explain the role of NAFLD in elevated RDW; meanwhile, RDW might be an important marker that reflects the inflammation in NAFLD patients. Therefore, the mechanism by which OEA could reduce RDW in our patients might be due to its anti-inflammatory activity and anti-atherosclerosis effect. However, in terms of other hematological parameters, there were no significant differences between the two comparing groups. Perhaps a longer intervention period or a higher dose of OEA is required for the favorable impacts of OEA on these parameters.

One of the strengths of the current trial is that patients with newly diagnosed NAFLD, not receiving treatment, were included in this study. Moreover, as far as we know, clinical trials examining the effects of OEA supplementation on atherogenic indices and hematological parameters in NAFLD patients are rare. In addition, weight reduction diet was presented for all the patients. Furthermore, a two-way ANCOA test adjusted for potential confounding variables was used for statistical analysis. However, some limitations should be considered in this study. First, serum levels of OEA were not measured due to funding constraints. The absence of liver biopsy for assessing NAFLD severity due to ethical considerations and patients’ refusal was another limitation of the current study.

## Conclusion

As this study showed that OEA supplementation associated with weight loss diet appears to have beneficial effects on lipid profile as well as RDW in obese patients with NAFLD, supplementation with OEA could be an effective therapeutic strategy for NAFLD. However, more well-designed clinical trials with different dosages of OEA and longer follow-up period are required.

## Acknowledgements

We sincerely thank the patients who participated in the present study.

## Funding

The study was financially supported by the Nutrition Research Center of Tabriz University of Medical Sciences, and Iran National Science Foundation (INSF).

## Competing interests

The authors declare no conflict of interest in publishing this paper. This study not supported by any grant money from a pharmaceutical company or for-profit organization.

## Ethical approval

The study was approved by the Ethics Committee of the Tabriz University of Medical Sciences, Tabriz, Iran (IR.TBZMED.REC.1397.694).

## Authors’ contribution

HT and AO designed the study; HT and FN drafted the manuscript and analyzed and interpreted the data; NF and AMK contributed to data collection; MSA contributed to the final revision of the manuscript. All authors critically revised the manuscript for important intellectual content and approved it for submission.

**Table 1:** General characteristics of study participants

	**OEA ( n =38)**	**Placebo ( n =38)**	***P***
Age (years)	41.73 (6.95)	39.27 (7.14)	0.597^a^
Gender			1.00^b^
Females	18 (47.37)	18 (47.37)	
Males	20 (52.63)	19 (52.63)	
Marital status			0.829^b^
Single	8 (21.05)	6 (15.79)	
Married	30 (78.95)	32 (84.21)	
Education			0.147^b^
Illiterate	2 (5.26)	7 (18.42)	
Diploma and lower	26 (68.42)	25 (65.79)	
Bachelors and higher	10 (26.32)	6 (15.79)	
Occupation			0.870 ^b^
Housewife	16 (42.11)	16 (42.11)	
Employee	14 (36.84)	16 (42.11)	
Self-employed	8 (21.05)	6 (15.78)	
Severity of fatty liver			1.00 ^b^
Mild	25 (65.79)	25 (65.79)	
Moderate	13 (34.21)	13 (34.21)	
Physical activity level			
Low	28 (73.67)	29 (76.33)	0.668^b^
Moderate	10 (26.33)	9 (23.67)	
TC (mg/dL)	180.51 (39.25)	185.05 (25.79)	0.558^a^
TG (mg/dL)	155.11 (54.51)	167.89 (60.86)	0.344^a^
LDL-C (mg/dL)	124.83 (28.47)	121.54 (21.16)	0.574 ^a^
HDL-C (mg/dL)	38.37 (6.64)	40.54 (6.59)	0.164 ^a^

TC, Total cholesterol; TG, Triglyceride; LDL-C, Low-density lipoprotein cholesterol; HDL-C, High-density lipoprotein cholesterol. Data presented as Mean (SD) for quantitative variable and number (%) for qualitative variables. ^a^ Independent samples *t* test. ^b^ Fisher's exact test.

**Table 2:** Atherogenic indices, blood pressure, and physical activity of the study participants throughout the study

	**OEA (n=38)**	**Placebo (n=38)**	**MD (95% CI),** ***P***
TC/HDL-C			
Baseline	4.94 (1.42)	4.49 (0.82)	0.45 (-0.08, 0.99), 0.101^b^
End	4.09 (0.89)	4.31 (0.92)	-0.18 (-0.65, 0.22), 0.452^c^
MD (95% CI), *P*^a^	-0.85 (-1.14, -0.56), **<0.001**	-0.18 (-0.43, 0.07), 0.162	
LDL-C/HDL-C			
Baseline	3.28 (0.88)	3.05 (0.74)	0.23 (-0.14, 0.60), 0.226^b^
End	2.81 (0.90)	2.72 (0.72)	0.45 (0.06, 0.85), **0.024**^c^
MD (95% CI), *P*^a^	-0.46 (-0.71, -0.22), **<0.001**	-0.33 (-0.53, -0.12), **0.003**	
TG/HDL-C			
Baseline	4.19 (1.76)	4.32 (1.85)	-0.13 (-0.96, 0.71), 0.762^b^
End	2.74 (1.14)	3.85 (1.66)	-1.06 (-2.06, -0.05), **0.039**^c^
MD (95% CI), *P*^a^	-1.45 (-1.87, -1.01), **0.006**	-0.47 (-1.03, 0.08), 0.095	
Non-HDL-C			
Baseline	142.13 (38.71)	144.51 (7.02)	-2.37 (-17.20, 12.44), 0.475^b^
End	127.59 (46.44)	135.24 (33.42)	-9.55 (-30.05, 10.94), 0.355 ^c^
MD (95% CI), *P*^a^	-14.54 (-624.69, -4.38), **<0.001**	-9.27 (-19.07, 0.53), 0.063	
Non-HDL-C/HDL-C			
Baseline	3.80 (1.22)	3.64 (0.72)	0.16 (-0.29, 0.63), 0.151^b^
End	2.61 (0.99)	3.38 (0.88)	-0.53 (-1.05, -0.02), **0.042**^c^
MD (95% CI), *P*^a^	-1.19 (-1.47, -0.90), **<0.001**	-0.26 (-0.49, -0.007), **0.044**	
BMI (kg/m^2^)			
Baseline	34.19 (3.24)	34.55 (4.19)	-0.33 (-1.87, 1.68), 0.872^b^
End	31.79 (4.19)	33.91 (3.45)	-2.11 (-1.86, -1.69), **<0.001**^d^
MD (95% CI), *P*^a^	-2.42 (-3.566, -1.88), **<0.001**	-0.64 (-0.97, -0.31), **<0.001**	
Energy (kcal)			
Baseline	2349.27 (814.66)	2258.39 (739.65)	18.95 (-361.55, 337.42), 0.815^b^
End	1564.33 (644.81)	1721.29 (699.11)	-377.62 (-533.49, -79.88), **0.011**^d^
MD (95% CI), *P*^a^	-784.93 (-1133.82, -618.49),**<0.001**	-537.11 (-687.53, -108.71), **0.012**	
SBP			
Baseline	119.74 (17.77)	114.62 (18.99)	5.12 (-4.81, 11.73), 0.641^b^
End	111.41 ( 19.37)	107.11 (14.73)	2.33 (-4.13, 8.03), 0.832^c^
MD (95% CI), *P*^a^	-8.33 (-12.11, -2.81),**0.037**	-7.51 (-13.33, -3.11), **0.006**	
DBP			
Baseline	71.96 (13.01)	76.59 (10.11)	-4.63 (-6.24, 11.31), 0.679^b^
End	69.93 (14.33)	72.41 (15.77)	-1.78 (-4.68, 8.15), 0.331^c^
MD (95% CI), *P*^a^	-2.03 (-6.31, 0.93), 0.146	-4.18 (-9.29, 0.84), 0.131	
PA (METs)			
Baseline	572.32 (196.72)	549.62 (199.60)	22.70 ( -69.05, 114.46), 0.623 ^b^
End	576.00 (189.50)	560.97 ( 206.12)	-7.36 (-23.16, 8.43), 0.356^C^
MD (95% CI), *P*^a^	3.68 (-3.94, 11.29), 0.335	11.35 (-2.69, 25.40), 0.110	

OEA, Oleoylethanolamide; TC/HDL-C, Total cholesterol/high-density lipoprotein cholesterol; LDL-C/HDL-C, Low-density lipoprotein cholesterol/ high-density lipoprotein cholesterol; TG/HDL-C, Triglyceride/ high-density lipoprotein cholesterol; Non-HDL-C, Non- high-density lipoprotein cholesterol; Non HDLc/HDLc, Non- high-density lipoprotein cholesterol/ high-density lipoprotein cholesterol; BMI, Body mass index; SBP, Systolic blood pressure; DBP, Diastolic blood pressure; PA, Physical activity; METs, Metabolic equivalents (MET-minutes/week). Mean (SD) and Mean Difference (95% CI) are presented for data. ^a^ Paired samples *t* test. ^b^ Independent samples *t* test. ^c^ ANCOVA test, adjusted for baseline values, age, changes in physical activity, energy intake, and BMI. ^d^ANCOVA test, adjusted for baseline values, age, and changes in physical activity.

**Table 3:** Hematological parameters of the study participants throughout the study

	**OEA (n=37)**	**Placebo (n=37)**	**MD (95% CI),** ***P***
WBC (10^3^/µL)			
Baseline	7.06 (1.44)	6.89 (1.29)	0.17 (-0.46, 0.80), 0.601^b^
End	7.04 (1.35)	6.96 (1.51)	0.37 (-0.40, 1.14), 0.343 ^c^
MD (95% CI), *P*^a^	-0.02 (-0.45, 0.40), 0.910	0.06 (-0.28, 0.42), 0.698	
Hb (g/dL)			
Baseline	14.31 (1.57)	14.50 (1.93)	0.18 (-1.00, 0.63), 0.656^b^
End	15.39 (1.60)	15.54 (1.98)	0.11 (-0.72, 0.48), 0.700^c^
MD (95% CI), *P*^a^	1.08 (0.84, 1.32), **<0.001**	1.04 (0.74, 1.35), **<0.001**	
RBC (10^6^/µL)			
Baseline	5.03 (0.43)	5.13 (0.44)	0.10 (-0.31, 0.09), 0.095^b^
End	5.14 (0.51)	5.12 (0.45)	0.12 (-0.06, 0.31), 0.188^c^
MD (95% CI), *P*^a^	0.11 (0.03, 0.18), **0.004**	-0.29 (-1.25, 0.65), 0.532	
HCT (%)			
Baseline	43.30 (4.10)	44.06 (4.89)	0.75 (-2.85, 1.33), 0.472^b^
End	44.77 (4.39)	44.92 (4.96)	0.85 (-2.39, 0.67), 0.267^c^
MD (95% CI), *P*^a^	1.46 (0.85, 2.08), **<0.001**	0.86 (0.09, 1.62), **0.028**	
MCV (FL)			
Baseline	85.04 (9.61)	86.15 (6.71)	0.75 (-2.85, 1.33), 0.472^b^
End	86.95 (10.05)	88.14 (5.39)	0.04 (-1.73, 1.64), 0.955^c^
MD (95% CI), *P*^f^	1.91 (1.22, 2.58), **<0.001**	1.98 (1.09, 2.88), **<0.001**	
MCH (pg)			
Baseline	28.65 (1.69)	33.02 (2.91)	0.17 (-0.92, 1.27), 0.752^b^
End	34.18 (1.67)	34.13 (6.04)	0.65 (-4.04, 2.73), 0.698^c^
MD (95% CI), *P*^f^	1.66 (1.30, 2.01), **<0.001**	3.18 (0.62, 5.72), **0.016**	
MCHC (g/dL)			
Baseline	32.95 (1.65)	33.01 (1.09)	0.06 (-0.71, 0.57), 0.837^b^
End	34.18 (1.82)	34.13 (2.29)	0.47 (-1.63, 0.68), 0.417^c^
MD (95% CI), *P*	0.29 (0.96, 1.48), **<0.001**	1.12 (0.46, 1.76), **0.001**	
RDW (%)			
Baseline	12.11 (0.67)	12.41 (1.15)	0.29 (-0.73, 0.14), 0.182^b^
End	11.63 (0.80)	12.08 (0.77)	0.28 (-0.56, -0.003), **0.041**^c^
MD (95% CI), *P*	0.48 (-0.70, -0.25), **<0.001**	0.32 (-0.58, -0.72), **0.014**	
PLT (10^3^/ µL)			
Baseline	270.35 (50.88)	258.02 (54.21)	12.32 (-12.04, 36.69), 0.317^b^
End	255.51 (49.90)	242.64 (55.59)	0.74 (-14.19, 15.68), 0.921^c^
MD (95% CI), *P*	14.83 (-19.61, -10.06), **<0.001**	15.37 (-25.27, -5.48), **0.003**	
MPV (FL)			
Baseline	8.02 (0.86)	7.79 (0.47)	0.23 (-0.09, 0.55), 0.157^b^
End	8.06 (0.81)	7.99 (0.49)	0.07 (-0.16, 0.31), 0.548^c^
MD (95% CI), *P*	0.04 (-0.07, 0.16), 0.456	0.19 (-0.08, 0.31), 0.141	
PDW			
Baseline	16.78 (0.60)	16.85 (0.65)	0.06 (-0.36, 0.22), 0.642^b^
End	16.63 (1.59)	16.94 (0.77)	-0.52 (-1.43, 0.38), 0.255^c^
MD (95% CI), *P*	0.16 (-0.33, -0.007), **0.041**	0.32 (-0.58, -0.07), **0.014**	

OEA, Oleoylethanolamide; WBC, White blood cell; Hb, Hemoglobin; RBC, Red blood cell; HCT, Hematocrit; MCV, Mean corpuscular volume; MCH, Mean corpuscular hemoglobin; MCHC, Mean corpuscular hemoglobin concentration; RDW, Red blood cell distribution width; PLT, Platelet; MPV, Mean platelet volume; PDW, Platelet distribution width. Mean (SD) and Mean Difference (95% CI) are presented for data. ^a^ Paired samples *t* test. ^b^ Independent samples *t* test. ^c^ ANCOVA test, adjusted for baseline values, age, changes in physical activity, energy intake, and BMI.
